# Case Report: Synchronous bilateral lipoma arborescens of the bicipitoradial bursa

**DOI:** 10.12688/f1000research.122432.1

**Published:** 2022-07-13

**Authors:** Rafik Elafram, Majdi Ben Romdhane, Nayssem Khessairi, Ahmed Hamdi

**Affiliations:** 1 Homeland Security Forces Hospital, Tunis Elmanar University, La Marsa, 2074, Tunisia

**Keywords:** benign, joint masses, lipoma arborescens

## Abstract

**Background**: Lipoma arborescens (LA) is an infrequent benign tumor made of mature sub-synovial fatty cell proliferation that may arise into the synovial joint, the bursae or the tendon sheaths. This condition affects mainly the knee joint, but the bicipitoradial bursa is an exceptional location. We report herein a case of a synchronous bilateral (LA) of the bicipitoradial bursa.

**Case presentation**: A 52-year-old patient, with no medical history, presented with a swelling of both front arms that had been progressing for nine years. Physical examination showed a mass in the antecubital fossae of 3cm on the left side and 0.5cm on the right side. Both masses were tender, well-defined, fixed, without inflammatory signs and painful on elbow flexion. A magnetic resonance imaging (MRI) scan was performed, revealing the presence of a septate soft-tissue mass of the distal portion of the brachial muscle of 70x46x27mm. This mass had a heterogeneous fat signal in its depth and a homogeneous fat composition on the outside. The diagnosis of liposarcoma was suspected. The patient underwent surgery to remove both masses. Gross examination showed a characteristic frond-like or digitiform pattern. Microscopical examination demonstrated papillary proliferation of the synovial villi. The final diagnosis was of LA. The patient had no complications and there was no recurrence of LA.

**Conclusions**: LA is a rare condition, and the bicipitoradial bursa is an exceptional location.

Histological confirmation is mandatory to correct the diagnosis.

## Introduction

Lipoma arborescens (LA) is an infrequent benign tumor made of mature sub-synovial fatty cell proliferation that may arise into the synovial joint, the bursae or the tendon sheaths. ^1^ Its name comes from *arbo* meaning tree, which describes the frond-line gross aspect of the mass. ^2^ It can also be named villous lipomatous proliferation of the synovial membrane thus indicating it’s non-neoplastic nature. ^3^ This condition affects mainly the knee joint, but the bicipitoradial bursa is an exceptional location. ^1^

We herein report a case of a synchronous bilateral (LA) of the bicipitoradial bursa. Extra or periarticular location of LA such as in our case in the bicipitoradial bursa are exceptional localization with few cases in the literature.

## Case presentation

A 52-year-old Caucasian male patient (occupation, policeman), with no medical history, presented with swelling of both front arms associated with cramps and pain irradiating to the shoulders, which had been developing for nine years.

There was no history of fever or weight loss nor traumatic accident. Physical examination showed a mass in the antecubital fossae of 3 cm on the left side and 0.5 cm on the right side. Both masses were tender, well-defined, fixed, without inflammatory signs and painful on elbow flexion. Ultrasound scan showed a fusiform under-aponeurotic intramuscular mass with lobulated edges, encasing the median nerve.

**Figure 1.**
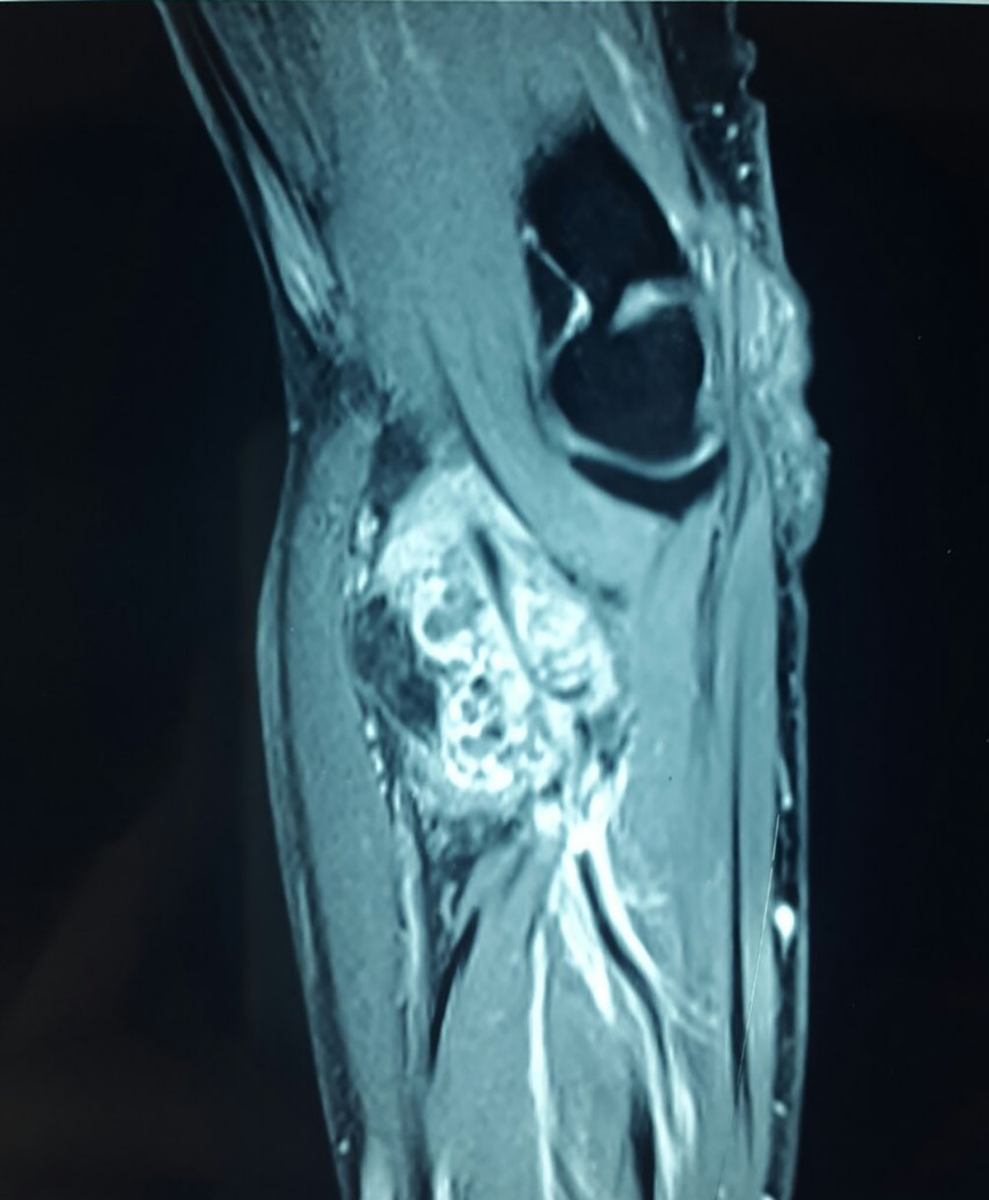
Sagittal magnetic resonance imaging (MRI) image of the left front arm with a lobulated complex mass with frond-like appearance extending from the bicipitoradial bursa.

**Figure 2.**
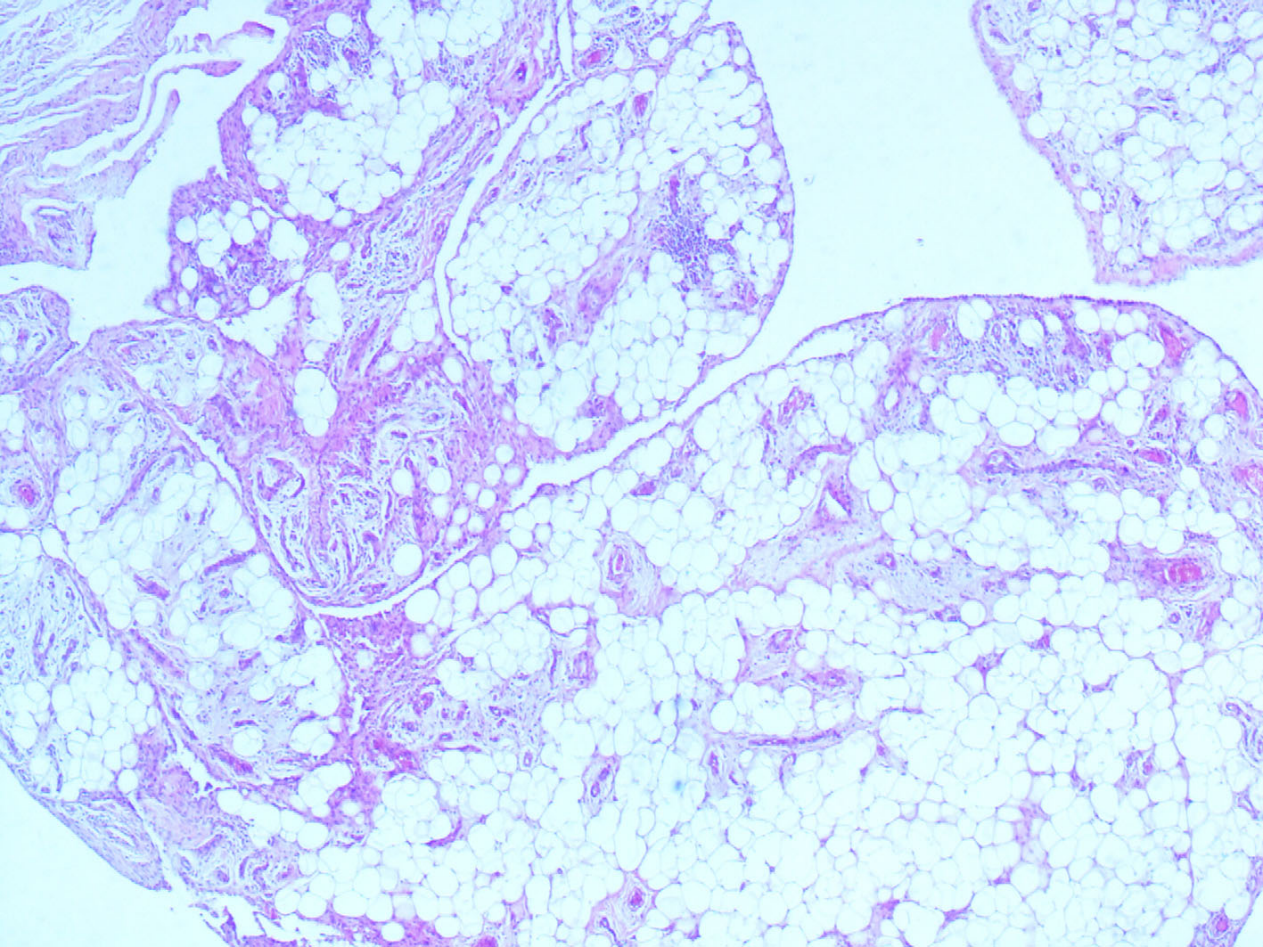
Lipoma arborescens (LA) pathology slide. Synovial villi enlarged by abundant mature adipocytes. Note the variable degrees of fatty infiltration of the subsynovial tissues (hematoxylin and eosin (HE); magnification, ×40).

**Figure 3.**
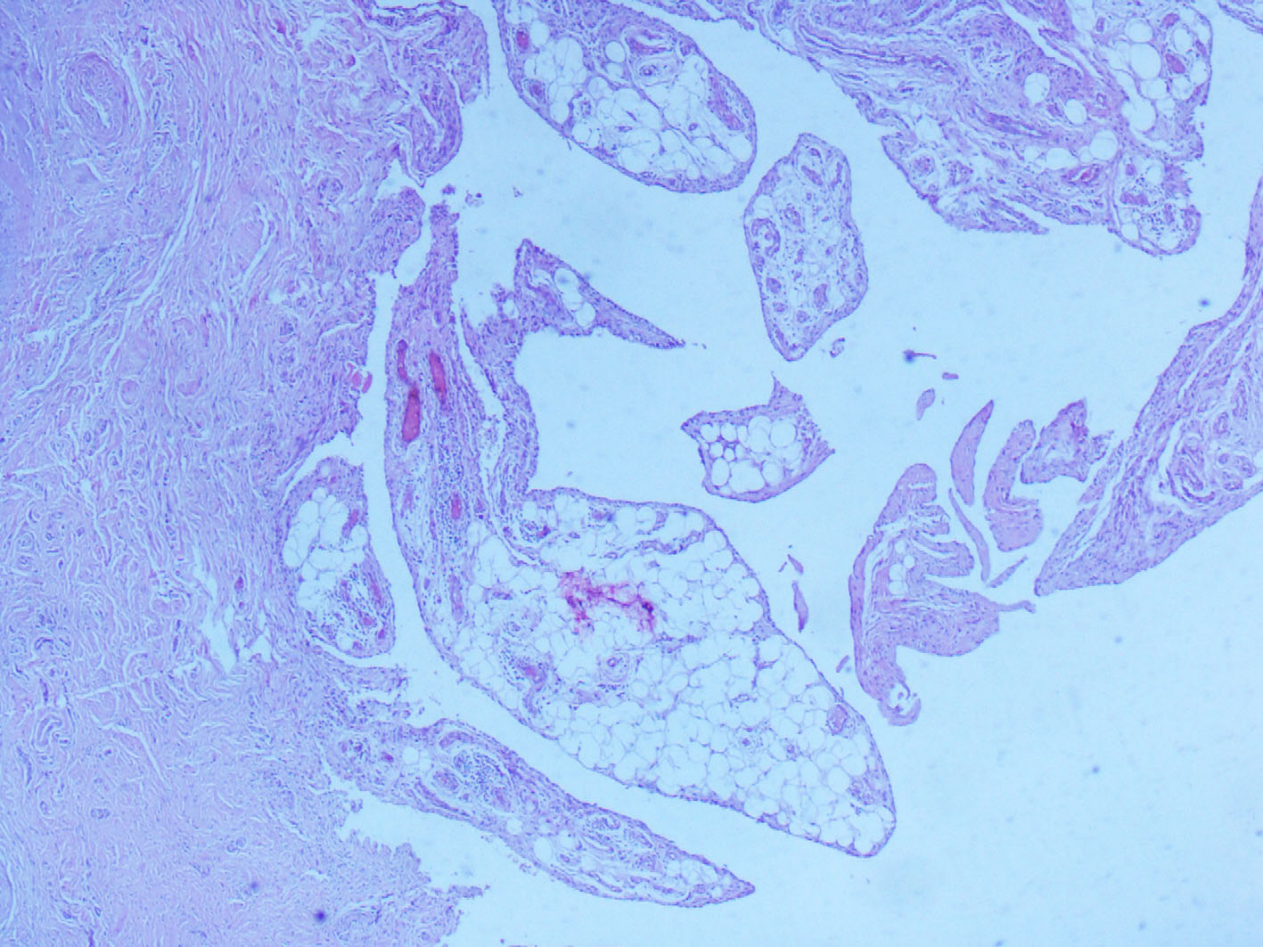
Lipoma arborescens (LA) pathology slide. Synovial villi enlarged by abundant mature adipocytes. Note the variable degrees of fatty infiltration of the subsynovial tissues (hematoxylin and eosin (HE); magnification, ×40).

**Figure 4.**
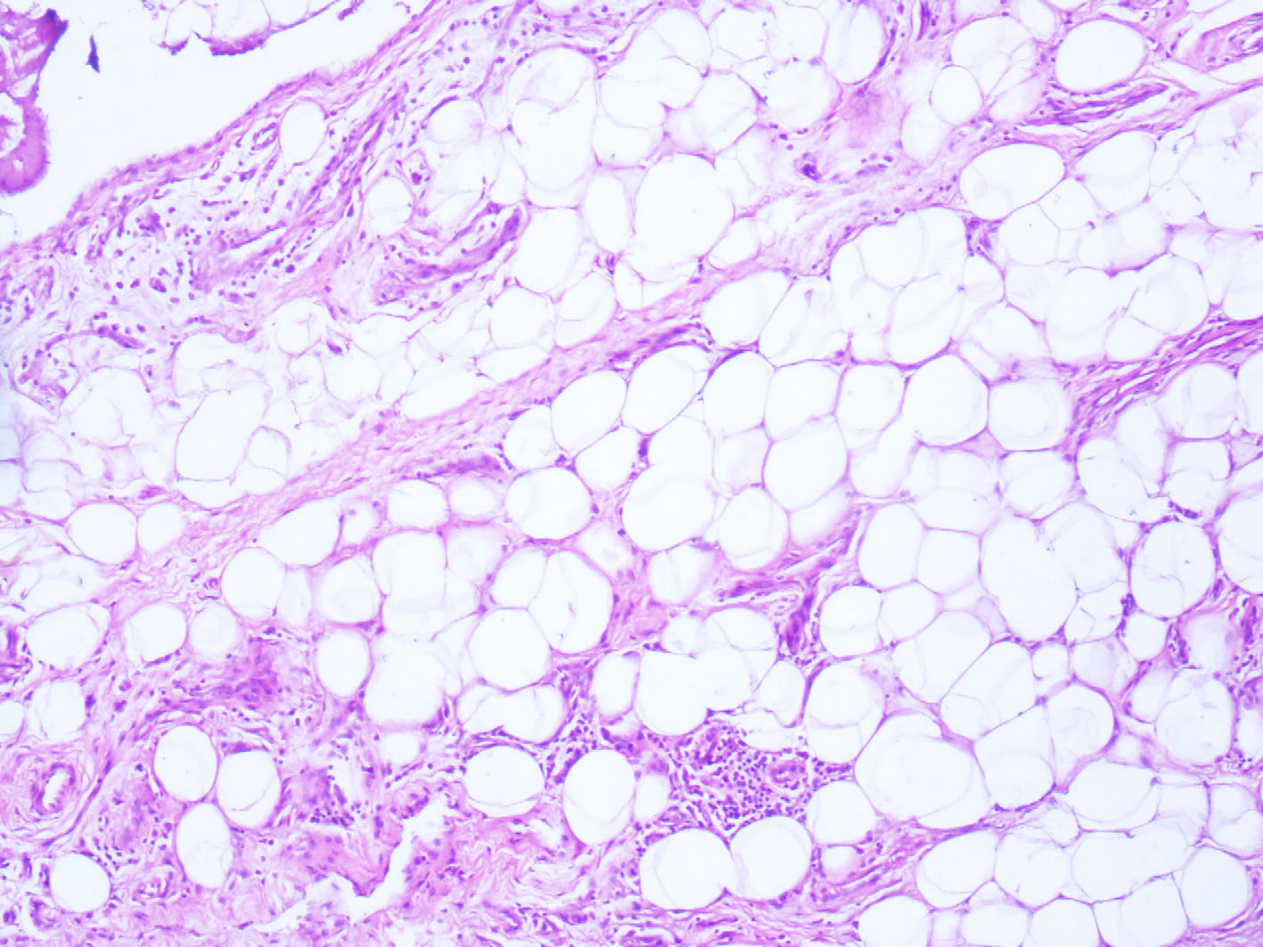
Lipoma arborescens (LA) pathology slide. The synovial layer is only a few cells with substitution of subsynovial tissue by mature adipocytes (hematoxylin and eosin (HE); magnification, ×200).

**Figure 5.**
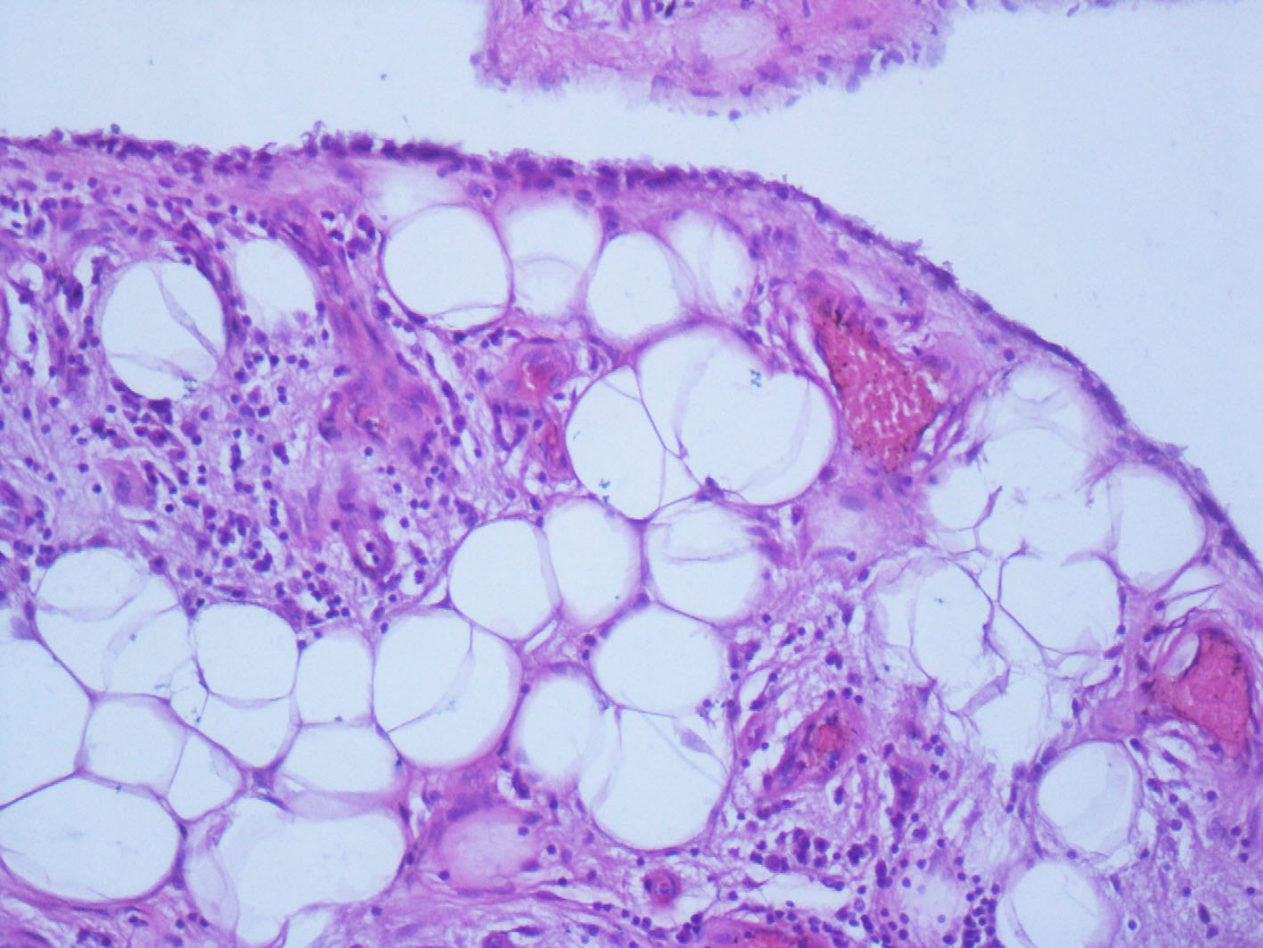
Lipoma arborescens (LA) pathology slide. Capillaries and small amounts of chronic inflammatory cells are present (hematoxylin and eosin (HE); magnification, ×400).

This aspect corresponded to a fibrolipoma of the median nerve without vascularization. A magnetic resonance imaging (MRI) scan was performed, revealing the presence of a septate soft-tissue mass of the distal portion of the brachial muscle of 70×46×27 mm ( Figure 1). This mass had a heterogeneous fat signal in its depth and a homogeneous fat composition on the outside. It was adjacent to the humerus and invaded the posterior cortical bone. These features led us to conclude the diagnosis of a liposarcoma. The patient had a biopsy of the left antecubital fossa. The histopathology concluded to a well-differentiated adipocytic tumor. The patient underwent surgery to remove both masses with complete removal of the tumors with no harm to the nervous system structure. Gross examination showed a characteristic frond-like or digitiform pattern. Microscopical examination demonstrated papillary proliferation of the synovial villi ( Figure 2 and Figure 3). These villi were covered by hyperplastic inflamed synovium with adipocyte axis, made of mature fatty cells and fine fibrovascular septa ( Figure 4 and Figure 5). The final histopathological examination confirmed the diagnosis of LA. The biopsy was not sufficient to confirm the diagnosis.

The post-operative follow-up over two years remained simple and quasi painless. The definitive histopathological examination of both masses led to the diagnosis of LA.

## Discussion

First described by Albert Hoffa in 1904, the incidence of LA is still unknown. Only a few cases have been reported in the literature. ^4^ In the reported cases, the ages range between 9 and 68 years old with no affinity for either sex. ^5^^,^^6^ Two types of LA have been described: primary and secondary, regarding to the age and the existence of associated conditions. The primary type occurs mainly in young patients during the first two decades of life and is idiopathic. Otherwise, the secondary type usually occurs in elderly patients and is associated with existing chronic damage such as degenerative disease, trauma, meniscal or synovial injury. This type is more commonly found. ^7^^,^^8^

LA is frequently observed in the knee, but can be described in the hips, shoulders, wrist, or elbow. The lesion mostly affects a single joint, but in some patients, it may be found in polyarticular form. Extra or periarticular location of LA such as in our case in the bicipitoradial bursa has rarely been reported. ^9^^,^^10^ Howe and Wenger have suggested that LA is divided into two groups: typical presentation (unilateral knee involvement) and atypical presentation (polyarticular LA and outside of the typical location of the knee). ^10^ Nevertheless, there was no significant difference in age of diagnosis, presence of degenerative or inflammatory arthritis between the typical and atypical presentations. ^10^ The etiology of LA is still unknown. Some hypotheses suggest that LA may appear in joints with chronic inflammation leading to proliferation and hypertrophy of the synovial membrane with subsequent adipose differentiation. The existence of a specific vulnerability of the sub-synovial fatty tissue has also been noted. ^9^^,^^11^ The role of steroid injections and diabetes has also been investigated. ^2^ Patients with LA usually present with painless joint swelling. ^8^ Some patients complain of an intermittent pain that can be attributed to the enclosure of the expanded joint villi. ^11^ The bicipitoradial bursa is situated between the distal tendon of the biceps brachii muscle and the anterior part of the tuberosity of the radius. It totally or partially surrounds the biceps tendon.

With pronation, the radius tuberosity rotates posteriorly, inducing a compression of the bursa between the tuberosity and the biceps tendon. If inflamed or distended, the bursa may cause an enclosure of the radial and the median nerves. By its localization, the bicipitoradial bursa is subject to pressure, which can explain the emergence of LA. Furthermore, the role of abnormal hypertrophy of the radial tuberosity has been described as increasing micro-trauma in high pressure areas, explaining the occurrence of LA in the bicipitoradial bursa. ^1^^,^^8^

In LA, results of laboratory tests, such as rheumatoid factors, uric acid, C-reactive protein and erythrocyte sedimentation rate, are normal. Arthroscopy in the affected joint can reveal a yellow-white hypertrophied synovium with adipocytic villi projections into the joint space. ^8^^,^^12^^,^^13^ Conventional radiographs have a limited value in this case, they only show nonspecific findings as in some cases articular soft tissue density is associated with signs of arthrosis. ^7^ LA appears on ultrasonography as a high echo pattern comparable with the adjacent subcutaneous adipocytic tissue. The consistency of the mass is mainly smooth and expandable. Ultrasonography is useful in detecting the localization and size of LA. ^3^ In our case, the ultrasonography imaging concluded to a fibro lipoma of the median nerve. Since the invention of MRI, diagnostic accuracy has increased. ^11^^,^^13^

LA usually presents as nodular or villous sites in hypersignal on T1 and T2 sequences that are erased on the short tau inversion recovery (STIR) or fat saturation sequences. The residual non-fatty component of the enlarged synovium in the condition shows heterogeneous hypersignals in T2 and STIR sequences and hypo signals or intermediate signals in T1 sequences. ^7^ However, in our case, the MRI findings were not unequivocal, and we were oriented to a neoplastic lesion. In fact, sometimes the imaging may imitate a well-diffracted liposarcoma (WDL). In cases of WDL, we can find a mostly adipose mass with an irregularly thickened nodular septa. ^14^ Most soft-tissue tumors can also manifest as neoplastic lesions. The decision of surgery is made if a tumor significantly grows in size or becomes symptomatic. ^15^

If preoperative investigations cannot firmly assess the benign, low-grade malignant or high-grade malignant nature of the lipomatous mass, then it is recommended that the patient undergoes a biopsy and two-stage removal of the tumor. ^16^ The gold standard treatment of LA is surgical excision. For LA, occurring in joints, arthroscopic synovectomy and open resection are the main interventions with identical results to open surgery and low recurrence rates. ^17^ Histopathological examination eventually confirmed the diagnosis of LA. Indeed, it presents as a fatty-mass with a finger-like or ramified villous proliferation. ^18^ Histologically, there is a synovial hyperplasia with scattered chronic inflammatory cells and extensive shedding of mature fat cells in the sub synovial tissue. ^9^^,^^18^

The fat cells are assisted by nourishing blood vessels and are overlayed by multiple synovial cells layers. Synovium responds to the irritation by increasing capillary blood flow, which lead to an influx of inflammatory cells, and synovial hyperproliferation. As this becomes chronic, adipocytes can also infiltrate the sub synovial tissue. ^8^

## Conclusions

LA is a rare condition and an infrequent cause of joint masses. The bicipitoradial bursa is an exceptional location. This entity has characteristic features, but radiological examination may miss the diagnosis especially in atypical locations. Recognizing the clinical and imaging findings is necessary to avoid misdiagnosing this condition.

## Data availability

### Underlying data

All data underlying the results are available as part of the article and no additional source data are required.

## Consent

Written informed consent for publication of their clinical details and clinical images was obtained from the patient.
