# Supplementary material for: Case Report: Synchronous bilateral lipoma arborescens of the bicipitoradial bursa
Source: F1000Res. 2022 Jul 13;11:787. [Version 1] doi: 10.12688/f1000research.122432.1 (PMC10907874; doi:10.12688/f1000research.122432.1)
Supplement: Peer review history

# Reviewer report: Reviewer response for version 1

**DOI:** 10.5256/f1000research.134418.r246639

**Published:** 2024-03-21

**Authors:** Sam Sedaghat

**Affiliations:** 1 University Hospital of Heidelberg, Heidelberg, Germany

This case report deals with a rare lipoma arborescence in the bicipitoradial bursa. Due to the rarity of this finding in an exceptional location, this case report is very interesting to a broad readership. I would advise the authors to perform a minor revision. My comment targets the main appearance and configuration of the tumor. As shown in Figure 1, the tumor has a lobulated configuration. Would be interesting to mention in the discussion that in future studies the main appearance and configuration of this tumor could be investigated and please cite the following two paper:[1],[2]. Although these papers mention soft tissue sarcoma, they show the importance of investigating the main appearance/configuration of benign and malignant soft tissue masses.

 Determining the configuration of such tumors is a quite novel approach. Therefore, these two papers are interesting for that approach I requested to add for further studies.

Are enough details provided of any physical examination and diagnostic tests, treatment given and outcomes?

Yes

Is the case presented with sufficient detail to be useful for other practitioners?

Yes

Is sufficient discussion included of the importance of the findings and their relevance to future understanding of disease processes, diagnosis or treatment?

Yes

Is the background of the case’s history and progression described in sufficient detail?

Yes

Reviewer Expertise:

MSK, soft tissue sarcoma

I confirm that I have read this submission and believe that I have an appropriate level of expertise to confirm that it is of an acceptable scientific standard.

## References

[1] : Configuration of soft-tissue sarcoma on MRI correlates with grade of malignancy. *Radiol Oncol* .2021;55(2) : 10.2478/raon-2021-0007 158-163 10.2478/raon-2021-0007 33600679 PMC8042815

[2] : Does the primary soft-tissue sarcoma configuration predict configuration of recurrent tumors on magnetic resonance imaging?. *Acta Radiol* .2022;63(5) : 10.1177/02841851211008381 642-651 10.1177/02841851211008381 33853376

# Reviewer report: Reviewer response for version 1

**DOI:** 10.5256/f1000research.134418.r246715

**Published:** 2024-03-01

**Authors:** Samuel Navarro, Emilio Medina-Ceballos

**Affiliations:** 1 university of Valencia, Valencia, Community of Valencia, Spain; 2 Pathology, Clinic Universitary Hospital, Valencia, Community of Valencia, Spain

Interesting case of an atypical lesion, bilateral and located in an unusual site.

 Arborescens lipoma is a well recognized clinico-pathological entity, maily found in the knee joint.. This teaching case reveals the importance of clinico-radiological approach ( discarding malignancies such as liposarcoma), and the mandatory confirmation with pathological analysis.

Are enough details provided of any physical examination and diagnostic tests, treatment given and outcomes?

Yes

Is the case presented with sufficient detail to be useful for other practitioners?

Yes

Is sufficient discussion included of the importance of the findings and their relevance to future understanding of disease processes, diagnosis or treatment?

Yes

Is the background of the case’s history and progression described in sufficient detail?

Yes

Reviewer Expertise:

Pathology of bone and soft tissue. Pediatric Pathology: tumoral.

We confirm that we have read this submission and believe that we have an appropriate level of expertise to confirm that it is of an acceptable scientific standard.

# Reviewer report: Reviewer response for version 1

**DOI:** 10.5256/f1000research.134418.r144562

**Published:** 2022-08-08

**Authors:** Reetu Kundu

**Affiliations:** 1 Department of Cytology & Gynecological Pathology, Postgraduate Institute of Medical Education and Research, Chandigarh, Punjab and Haryana, India

The authors have described a rare entity. The report of a case of a synchronous bilateral lipoma arborescens of the bicipitoradial is uncommon. The bicipitoradial bursa is indeed an exceptional location. As pointed out recognizing the clinical and imaging findings is necessary to avoid misdiagnosing this condition.

 One suggestion is to improve the contrast for Figures 2 and 3.

Are enough details provided of any physical examination and diagnostic tests, treatment given and outcomes?

Yes

Is the case presented with sufficient detail to be useful for other practitioners?

Yes

Is sufficient discussion included of the importance of the findings and their relevance to future understanding of disease processes, diagnosis or treatment?

Yes

Is the background of the case’s history and progression described in sufficient detail?

Yes

Reviewer Expertise:

Cytology and Gynecological Pathology

I confirm that I have read this submission and believe that I have an appropriate level of expertise to confirm that it is of an acceptable scientific standard.
